# Energy Efficient Forward Osmosis to Maximize Dewatering Rates

**DOI:** 10.3390/membranes15060171

**Published:** 2025-06-07

**Authors:** Jongmin Jeon, Dongkeon Kim, Suhan Kim

**Affiliations:** 1 R&D Institute, Hyorim E&I, 20 Gukgasandan-daero 40 beon-gil, Dalseong-gun, Daegu 43008, Republic of Korea; jongmin151@gmail.com; 2 Department of Civil Engineering, Pukyong National University, 45 Yongso-ro, Nam-gu, Busan 48513, Republic of Korea; dongkk7823@gmail.com

**Keywords:** forward osmosis (FO), draw solution split distribution (DSSD), energy efficiency, dewatering, membrane module configuration, water reuse, resource recovery

## Abstract

Forward osmosis (FO) is a membrane separation process driven by the osmotic pressure difference between a high-salinity draw solution (DS) and a low-salinity feed solution (FS). This pressure-free dewatering method is highly energy efficient, making it suitable for concentration and resource recovery. However, conventional FO systems using series-connected modules suffer from progressive DS dilution and FS concentration, leading to a reduction in the osmotic driving force and thereby limiting the overall performance. To address this issue, we propose a novel hybrid FO module configuration in which the FS flows in series while the DS is split and distributed in parallel across moules. This configuration was evaluated using an experimentally validated FO module model and RO simulation tools. Under seawater (600 mM NaCl) as DS and brackish water (10 mM NaCl) as FS, a conventional three-stage FO module achieved an enrichment ratio of 2.5 with an energy consumption of 0.151 kWh/m^3^. In contrast, the proposed draw solution split distribution (DSSD) achieved an enrichment ratio of 12.5 at a reduced energy consumption of 0.137 kWh/m^3^. In comparison, a reverse osmosis system consuming 0.58 kWh/m^3^ achieved a similar enrichment ratio of 12.3. These results demonstrate the high energy efficiency and dewatering capacity of the proposed FO configuration, highlighting its potential for industrial applications in food processing, beverage production, pharmaceuticals and agriculture.

## 1. Introduction

Forward osmosis (FO) is a membrane separation process in which water molecules migrate from a low-salinity feed solution (FS) to a high-salinity draw solution (DS) across a semi-permeable membrane driven by an osmotic pressure difference. Since FO operates under minimal hydraulic pressure, it enables the energy-efficient concentration of FS or dilution of DS under low-pressure conditions [1,2].

In addition to energy efficiency, FO is gaining attention in the field of wastewater treatment and resource recovery due to its potential to concentrate FS and facilitate the selective extraction of valuable solutes. For instance, Mo et al. [3] demonstrated lithium recovery from spent lithium-ion battery wastewater using FO, while Almoalimi and Liu [4,5,6] reported recovery of phosphorus, calcium, and magnesium from concentrated municipal effluents. Volpin et al. [7] also proposed an FO-based approach for recovering nitrogen and phosphorus from urine, with several other studies further supporting FO’s potential in nutrient recovery from urine-based wastewater [8,9,10,11]. Cath et al. (2006) demonstrated the applicability of FO for the low-temperature concentration of heat-sensitive beverages such as fruit juices and coffee extracts [12]. Holloway et al. [13] applied FO for the concentration and purification of antibiotics in pharmaceutical processes. Zhao et al. [14] reported the separation of organic compounds from high-strength industrial wastewater using FO, highlighting its potential for chemical manufacturing and wastewater minimization.

To realize the practical implementation of FO technology, scaling up through the connection of multiple FO modules in a multistage configuration is essential. Although several studies have explored commercially available FO modules, only a limited number of experimental case studies at the module scale have been reported to date. Xu et al. [15] evaluated the performance of the first commercial FO module by HTI, the Hydrowell module (active membrane area: 0.94 m^2^), under varying crossflow rates and draw solution concentrations. Phuntsho et al. [16] conducted experiments using the 4040MS module (active membrane area: 3.2 m^2^) developed by Hydration Technologies, examining the effects of crossflow velocity, pressure, temperature, and different draw solutes. Kim et al. [17] tested two 8-inch spiral-wound FO modules: the CTA module by HTI (9 m^2^) and the thin-film composite module by Toray Industries, Korea (15 m^2^), under varying crossflow conditions, solution concentrations, and hydraulic pressures. Kim et al. [18] further evaluated the performance of a four-stage serial configuration using the TFC 8-inch spiral-wound FO module (active membrane area: 15 m^2^) from Toray Chemical Korea. The experiments began with FS and DS flow rates of 5 and 40 L/min, respectively. As the stages progressed, the DS became increasingly diluted and the FS more concentrated, leading to a decrease in osmotic pressure and a corresponding drop in flux from 25 LMH (L/m^2^h) to 13.5 LMH.

Ali et al. [19] developed a forward osmosis system design software based on performance data obtained from the FO8040 module (active membrane area: 15.3 m^2^) by Toray Chemical Korea and subsequently applied it to the design of a large-scale 100,000 m^3^/day FO system. Field et al. [20] conducted experiments using the SepraMem 4040 FO spiral element (active membrane area: 1.6 m^2^) by HTI (Albany, OR, USA) to evaluate the effects of different spacer types and feedwater sources, including sodium chloride (NaCl) based solutions and NEWater brine. Jeon et al. [21] investigated the performance of an 8-inch spiral-wound FO module by Toray Chemical Korea to derive characteristic parameters for FO module modeling. Song et al. [22] performed experiments on a plate-type FO module (active membrane area: 7 m^2^) by Porifera, focusing on variations in crossflow velocity and draw solution concentration. Im et al. [23] evaluated the performance of FO modules for use in a FO-RO hybrid system cost analysis, conducting experiments with both a 4-inch spiral-wound FO module by Toray Chemical Korea (active membrane area: 3.1 m^2^) and a plate-type module by Porifera.

In a serially connected FO system, the dilution of the DS and concentration of the FS progressively reduce the osmotic pressure difference, thereby weakening the driving force and diminishing the performance of downstream modules. This phenomenon ultimately limits the overall efficiency of the system. Manufacturers have also recommended maintaining the FS pressure higher than the DS pressure during operation to prevent potential damage to the glue lines of the membrane module, which can occur if excessive pressure is applied to the DS channel [24]. Furthermore, in spiral-wound FO modules, the DS flow path is structurally more complex than that of the FS, resulting in a greater head loss. As water permeates from the FS to the DS, the flow rate in the DS channel increases toward the downstream end, exacerbating the head loss accumulation [25]. These hydraulic constraints suggest that the maximum number of modules that can be connected in series may be limited to fewer than four modules.

To overcome the limitations associated with conventional serially connected FO systems, this study proposes a novel module configuration strategy termed draw solution split distribution (DSSD). In this configuration, the DS is distributed in parallel to each module while the FS continues to flow in series. By supplying concentrated DS individually to each module, the osmotic driving force can be maintained throughout the system, and the issue of cumulative head loss in the DS channel can be effectively addressed. This study aimed to experimentally and numerically evaluate the performance of the proposed DSSD in terms of energy efficiency and dewatering capability and to compare its performance with both conventional serial FO configurations and reverse osmosis (RO) systems in terms of concentration ratio and energy consumption.

## 2. Materials and Methods

### 2.1. Draw Solution Split Distribution

#### 2.1.1. Limitations of Serially Connected Draw Solutions

**Figure 1:**
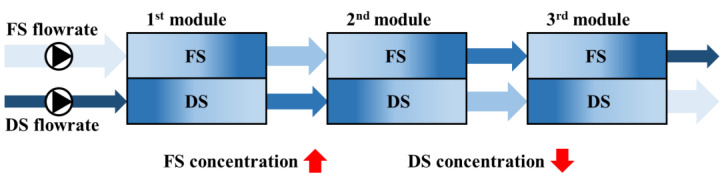
Conceptual diagram of FO process with serially connected modules.

As illustrated in Figure 1, in the serial configuration of the FO process, a single DS and FS are passed sequentially through the FO modules. During this process, the DS progressively diluted while the FS became increasingly concentrated. As a result, the osmotic pressure gradient between the DS and FS decreased significantly downstream. In these downstream modules, the reduced driving force leads to a significant decline in water flux, ultimately lowering the overall water recovery and energy efficiency of the system as a whole.

In full-scale FO systems employing spiral-wound modules, the flow characteristics of the FS and DS, as well as the channel geometry, become significantly more complex than those in lab-scale coupon tests. These complexities intensify both internal and external concentration polarization phenomena and introduce design challenges, such as flow maldistribution and dead zone formation. As a result, the water flux is further limited in practical applications.

#### 2.1.2. Concept of Draw Solution Split Arrangement

To address the limitations of the conventional serial configuration, a novel connection strategy, the DSSD, has been proposed. Instead of supplying the entire DS stream sequentially through the FO modules, the DSSD configuration divides the DS flow and delivers it individually to each module. This approach helps maintain a more consistent osmotic pressure gradient across each module, thereby improving the overall system performance.

The DSSD serves not only as a modification of flow routing but also as an effective strategy to address spatial flux imbalances across the system. First, by maintaining a relatively consistent DS concentration at the inlet of each module, the system ensured a more uniform flux and stable performance throughout all modules. Additionally, the dilution rate of the DS can be better controlled, preventing the decline in the osmotic driving force that typically occurs in serial configurations. Consequently, this approach enhances the average water flux and, in the long term, contributes to improved overall water recovery.

Moreover, reducing the flux deviations among the modules enhances operational stability. Sudden drops in flux or pressure imbalances within specific sections can impose mechanical stresses on the system. By adopting the DSSD configuration and supplying the draw solution individually to each module, these risks can be effectively mitigated. This not only improves the treatment performance but also contributes to extending the membrane lifespan and reducing the cleaning frequency.

**Figure 2:**
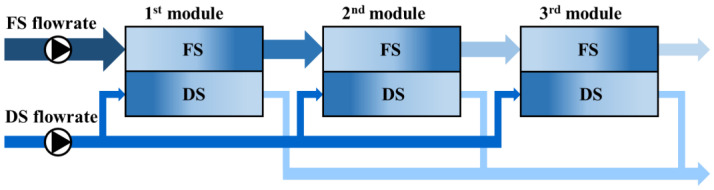
Conceptual diagram of the DSSD configuration in the FO process.

Figure 2 illustrates the concept of the proposed DSSD configuration. The DS was split at three points and fed in parallel to each FO module, with each DS stream collected separately after passing through its respective module. This design allows for the maintenance of a high osmotic driving force by minimizing the DS dilution, thereby sustaining the water flux throughout the system.

In addition, Jeon et al. [25] reported the phenomenon of flux saturation in spiral-wound FO modules, where the water flux no longer increased beyond a certain point due to the flow complexity within the DS channel. This finding underscores the structural limitations of using a single DS configuration. The DSSD approach mitigates these flow-induced constraints by distributing the DS to each module, thereby enhancing hydrodynamic stability and maintaining a favorable concentration gradient at the membrane surface.

The conventional approach of supplying DS in a single serial flow presents structural limitations, particularly as system performance declines with an increasing number of modules. To overcome this constraint, the DSSD strategy has emerged as a promising alternative, offering both enhanced performance and improved operational flexibility. This study investigates the effectiveness of the DSSD configuration through process design and performance evaluation based on the proposed concept.

### 2.2. Forward Osmosis Process Design

In this study, an FO module model developed by our research team was utilized [22]. Due to the structural complexity of spiral-wound FO modules, discrepancies often arise between theoretical predictions and experimental results. To address this issue, a simplified yet reliable performance prediction model was developed for a commercial 8-inch spiral-wound FO module (FO8040, Toray Chemical Korea Inc., Seoul, Republic of Korea).

The initial model was developed as a module-averaged system based on internal concentration polarization (ICP) and external concentration polarizations (ECP), with flow, concentration, and pressure variables simplified into a one-dimensional framework.

In this module-averaged framework, the flow, concentration, and pressure variables were treated as averaged values across the membrane leaf. The effective water flux (*J_w_*) and reverse salt flux (*J_s_*) were calculated as follows:

(1) $$J_{w}=A\cdot \left(∆\pi_{eff}-∆P\right)$$

(2) $$J_{s}=B\cdot \frac{c_{d}-c_{f}}{∆\pi_{eff}}$$

where *A* and *B* are the water and salt permeability coefficients; Δ*π*_eff_ is the effective osmotic pressure difference accounting for ICP and ECP; and Δ*P* is the transmembrane pressure. The structural parameter *S* used in the ICP term was determined using FO coupon tests. ICP resistance was modeled using the following equation:

(3) $$K_{ICP}=\frac{S}{D}$$

The mass transfer coefficient *k* was estimated using the Sherwood number (*Sh*) and diffusion coefficient (*D*) correlations for turbulent flow:

(4) $$SH=0.04{Re}^{0.75}{Sc}^{0.33}$$

(5) $$k=\frac{D\cdot Sh}{D_{H}}$$

where *Re* and *Sc* are the Reynolds and Schmidt numbers, and *d_H_* is the hydraulic diameter based on the spacer-filled channel geometry.

A total of 12 module-scale experiments (116 data points) using deionized water as the FS and NaCl as the DS were used to validate the model. Initial simulations showed a normalized root mean square error (NRMSE) of ~35%, which was primarily attributed to non-ideal phenomena, such as DS channel compression under elevated Δ*P*. As demonstrated by Jeon et al. [21], increased transmembrane pressure reduces the DS channel height, thus lowering the ECP and increasing the flux. Incorporating this effect into the model via a pressure-corrected fitting term significantly improved the prediction accuracy, reducing the NRMSE to ~10%.

The final model incorporated Δ*P* and C_d_⋅Δ*P* as the empirical correction terms. This model was validated to be accurate and applicable for full-scale process design with relatively low experimental data requirements (~30 points).

### 2.3. Forward Osmosis Module Experiment

In this study, the operational performance of a spiral-wound FO module was experimentally evaluated, and the results were compared with the predictions of a developed numerical model. To facilitate this, an experimental system capable of analyzing water flux and reverse salt flux was constructed using a full-scale FO module.

The experimental setup consisted of two circulation loops for the FS and DS, each connected to a 400 L storage tank, centrifugal pump, flowmeter, pressure gauge, and temperature control unit. The FO module (FO8040, Toray Chemical Korea Inc., Republic of Korea, 15.3 m^2^ membrane area) was installed in a standard 8-inch pressure vessel. In this study, *A*, *B*, and *S* of FO8040 were 1.59 × 10^−11^ m/sPa, 3.87 × 10^−7^ m/s, and 280 μm, respectively, at 18 °C [22].

**Figure 3:**
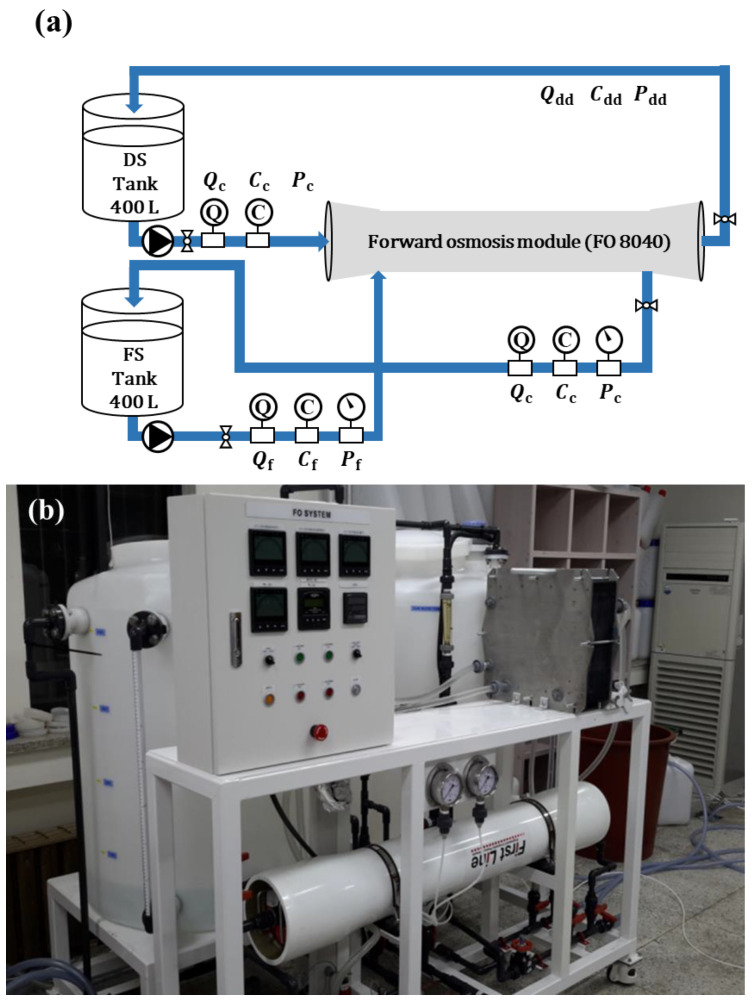
(**a**) Flow diagram of FO module (**b**) the actual photograph of FO module system.

The flow rates and pressures were continuously monitored, and data were recorded at fixed intervals. The hydraulic layout is illustrated in Figure 3a, and the physical setup is shown in Figure 3b. The FS and DS were supplied via separate circuits, enabling individual control of the flow and operating conditions across the membrane.

The experiments were conducted using a module-scale FO test unit equipped with an 8-inch spiral-wound module. Figure 3a illustrates the flow configuration of the system. In the diagram, *Q*, *C*, and *P* represent the flow rate, concentration, and pressure, respectively, while the subscripts *f*, *c*, *d*, and *dd* indicate the FS, concentrated FS, DS, and diluted DS, respectively.

During the operation, the FS and DS were supplied from and recirculated to separate 400 L water tanks. As the process proceeded, the FS gradually became concentrated while the DS was progressively diluted. The flow rates (*Q_f_*, *Q_c_*, and *Q_d_*), concentrations (*C_f_*, *C_c_*, *and C_d_*), and pressures were continuously monitored using sensors integrated into the system. In contrast, the flow rate (*Q_dd_*) and concentration (*C_dd_*) of the diluted draw solution were not measured directly but calculated using material balance equations (Equations (6) and (7)) based on the internal module flow dynamics.

(6) $$Q_{dd}=Q_{d}+(Q_{f}-Q_{c})$$

(7) $$C_{dd}=\frac{\left(Q_{d}C_{d}+Q_{f}C_{f}-Q_{c}C_{c}\right)}{Q_{dd}C_{dd}}$$

The feed water used in the experiments was produced by treating tap water with a 4-inch RO membrane (RE4040-SHN, Toray Chemical Korea Inc., Seoul, Republic of Korea). To remove residual chlorine from the tap water, sodium bisulfite (NaHSO_4_) was added as a pretreatment step. For DS preparation, industrial-grade purified sodium chloride (NaCl) supplied by OCI Co., Ltd. (Shanghai, China) was used to adjust the concentration. All experiments were conducted at a controlled temperature of 18 °C. To prevent cross-contamination between tests and maintain membrane performance, each experimental set was followed by a cleaning procedure using purified water prior to the next run.

To evaluate the hydraulic head loss associated with the flow characteristics of the FS and DS within the membrane module, the pressures at the inlets and outlets of both streams, *P_f_*, *P_c_*, *P_d_*, and *P_dd_*, were measured using pressure sensors. The water flux (*J_w_*) and reverse salt flux (*J_s_*) were calculated using Equations (8) and (9). To investigate the module performance under various operating conditions, flow rates and pressures were adjusted by adjusting the valves installed at the module inlet and outlet.

(8) $$J_{w}=\frac{\left(Q_{f}-Q_{c}\right)}{A_{m}}$$

(9) $$J_{s}=\frac{\left(Q_{c}C_{c}-Q_{f}C_{f}\right)}{A_{m}}$$

Among them, *Q_f_* and *C_f_* represent flow rate and salt concentration of the FS, respectively, while *Q_c_* and *C_c_* correspond to those of the concentrated feed stream. *A_m_* refers to the active membrane area used in the experiments.

### 2.4. Calculation Method for Energy Consumption

In this study, the energy consumption of both FO and RO processes was quantitatively evaluated by applying energy calculation equations that incorporate flow rate, pressure, and pump efficiency for each process. The energy consumption was determined using Equation (5), where all variables were evaluated using a combination of simulation data from the design program and experimentally measured values.

**Figure 4:**
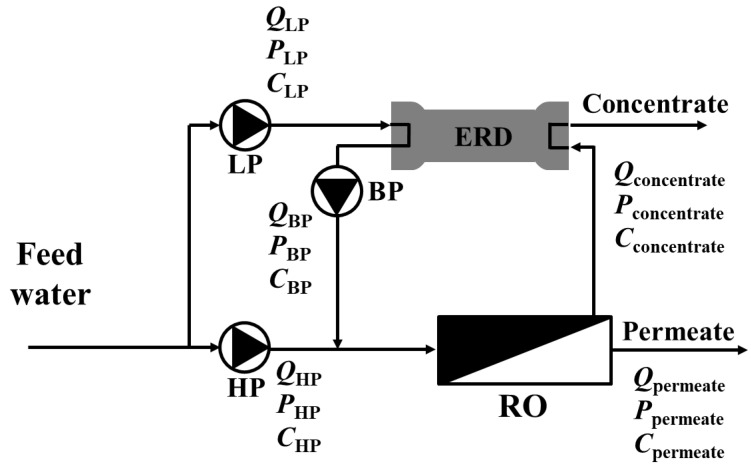
A schematic diagram of the RO process.

A schematic diagram of the RO process is shown in Figure 4, and the specific energy consumption per cubic meter of treated water was calculated using Equation (10) [26].

(10) $$E=\frac{\frac{Q_{HP}P_{HP}}{\eta_{HP}}+\frac{Q_{LP}P_{LP}}{\eta_{LP}}+\frac{Q_{BP}P_{BP}}{\eta_{BP}}}{36Q_{p}}$$

In Equation (5), *E* represents the specific energy consumption (kWh/m*^3^*), *Q* is the flow rate (m*^3^*/d), *P* indicates the pressure (bar), and η refers to the efficiency (%) of the pumps and energy recovery devices. *HP*, *LP*, and *BP* indicate the high-pressure, low-pressure, and booster pumps, respectively. The LP is responsible for delivering water to the energy recovery device (ERD), while the BP increases the pressure of the stream pressurized by the ERD to match that of the HP. The efficiency of the ERD was assumed to be 90%, and the efficiencies of the pumps and motors were obtained using the Power Model Pro software developed by Energy Recovery, Inc., San Leandro, CA, USA. [27]. The flow rate, pressure, and other parameters required for calculating the energy consumption of the RO process were obtained from the simulation results using the WAVE software developed by DuPont (Wilmington, DE, USA). Similar to the RO process, the energy consumption of the FO process was calculated based on the product of the flow rate and pressure across the feed solution pump and draw solution pump, taking into account the respective efficiencies of each pump. In this study, Equation (10) was adopted to calculate the specific energy consumption based on a simplified system-level approach and validated through DuPont WAVE simulations and experimental measurements. Although this formulation is widely used for comparative system analysis, it inherently assumes simplified hydrodynamics.

## 3. Results

### 3.1. Serial Arrangement of Forward Osmosis Process

**Figure 5:**
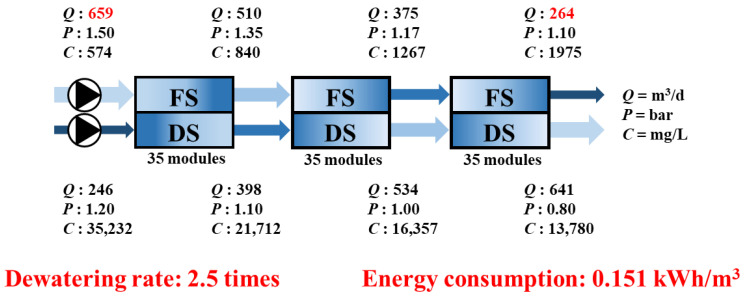
Impact of serial FO design on system performance and energy consumption.

As previously discussed, the serial configuration of FO modules results in the progressive dilution of the DS and concentration of the FS, which gradually reduces the osmotic pressure difference across the membrane. This decline in the driving force ultimately limits *J*_w_, especially in the downstream modules. A simulation of the FO process with a serial module configuration was performed to quantitatively verify the theoretical limitation. The results of the simulation are shown in Figure 5.

To simulate a serially connected module configuration, a single FO module was operated stepwise, with the output from each stage serving as the feed for the subsequent stages.

Through the serial configuration, the dewatering performance, represented by the concentration factor of the FS, was approximately 2.5, and the specific energy consumption was calculated to be 0.151 kWh/m^3^. Although this suggests an energy efficiency advantage over conventional RO processes, further performance enhancement is limited by structural constraints, such as declining *J*_w_ and cumulative dilution of the DS.

These experimental results serve not only to quantitatively verify the extent to which the serial configuration can be effective in FO processes but also to provide a baseline for comparison with the improved performance expected from the DSSD proposed in Section 2.1.

### 3.2. Draw Solution Split Arrangement of Forward Osmosis Process

#### 3.2.1. Experimental Verification of Forward Osmosis Performance Using Draw Solution Split Module Arrangement

**Figure 6:**
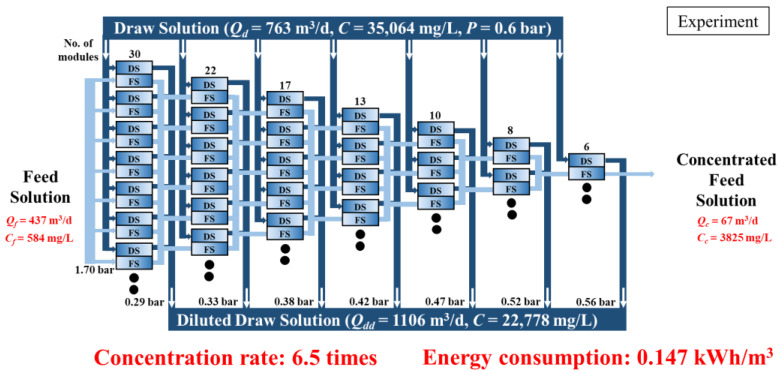
Experimental results of the DS-split FO module arrangement.

Figure 6 illustrates the maximum concentration performance achieved based on the experimental results using the DS-split configuration in the FO process. As described in Section 3.1, the experiment was conducted stepwise using a single FO module, considering the number of parallel units at each stage. In this setup, the FS had an inlet flow rate (*Q*_f_) of 437 m^3^/d with a concentration (*C*_f_) of 584 mg/L, corresponding to 10 mM NaCl. The DS was supplied at a rate of 763 m^3^/d with a concentration (*C*_d_) of 35,064 mg/L (600 mM NaCl). The initial pressures of the DS and FS were 0.6 and 1.7 bar, respectively. The black dots in the figure indicate that there are more membrane modules in actuality, which are omitted from the figure for brevity.

As the FO process progressed, water permeation through the membrane caused a reduction in the FS flow rate, while the DS was introduced in parallel to each module. Accordingly, the number of modules in parallel was gradually reduced to maintain a nearly constant flow rate per module on the FS side. The head loss was reduced owing to the application of the DSSD strategy, in which the DS was supplied in parallel to each module, and the corresponding decline in *J*_w_ in the downstream modules. As a result, the DS outlet pressure gradually increased from 0.29 to 0.56 bar.

As a result, the FS flow rate was concentrated from 437 m^3^/d to 62 m^3^/d, and the NaCl concentration increased from 584 mg/L to 3825 mg/L, corresponding to a concentration factor of 6.5. The average *J*_w_ was approximately 9.5 LMH, and the total specific energy consumption was calculated to be 0.147 kWh/m^3^, comprising 0.094 kWh/m^3^ from FS pumping and 0.053 kWh/m^3^ from DS pumping.

#### 3.2.2. Simulation-Based Verification of Forward Osmosis Performance Using Draw Solution Split Module Arrangement

**Figure 7:**
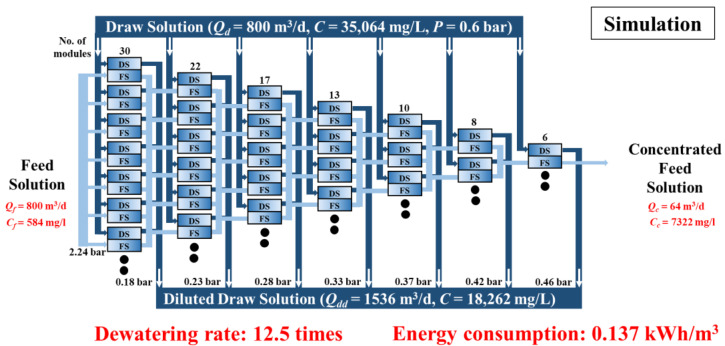
Simulation results of the DS-split FO module arrangement.

Figure 7 presents the simulation results for maximizing the concentration performance using the FO process with the DSSD configuration. In this scenario, both FS and DS were supplied at a rate of 800 m^3^/d. The FS concentration (*C*_f_) was 584 mg/L (10 mM NaCl), and the DS concentration (*C*_d_) was 35,064 mg/L (600 mM NaCl). The initial pressures were 2.24 bar for FS and 0.6 bar for DS. The black dots in the figure indicate that there are more membrane modules in actuality, which are omitted from the figure for brevity.

To maintain consistent flow per unit despite decreasing FS flow, the number of parallel DS channels was gradually reduced. This approach minimized the head loss and resulted in an increase in the DS outlet pressure from 0.18 to 0.46 bar.

As a result, the FS was concentrated from 800 m^3^/d to 64 m^3^/d, and the NaCl concentration increased from 584 mg/L to 7322 mg/L, achieving a concentration factor of 12.5. The average *J*_w_ was approximately 18.9 LMH, and the total specific energy consumption was calculated to be 0.137 kWh/m^3^, comprising 0.108 kWh/m^3^ from FS pumping and 0.029 kWh/m^3^ from DS pumping.

Compared with the experimental results, the simulated case exhibited lower energy consumption. Although the FS-side energy usage was higher owing to the increased flow rate and operating pressure, the overall energy demand was reduced owing to the higher *J*_w_ achieved, which lowered the DS-side energy consumption. As described in Equation (5), the specific energy consumption is inversely proportional to the amount of water produced; hence, a higher *J*_w_ leads to greater water recovery and improved energy efficiency.

### 3.3. Comparison with Reverse Osmosis Simulation Results

**Figure 8:**
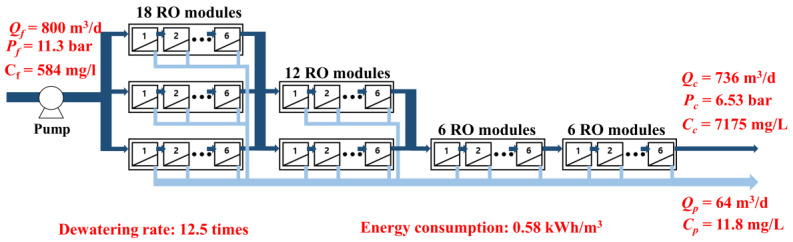
Simulation results of the reverse osmosis process.

To compare the maximum concentration condition of the FO process using the DSSD (Figure 7), an RO process simulation was conducted, as shown in Figure 8. The RO membrane module used in the simulation was BW30HR-440i from DuPont, with an active membrane area of 41 m^2^. The feed flow rate and concentration were set to 800 m^3^/d and 584 mg/L (10 mM NaCl), respectively. The simulation indicated that 42 RO membrane modules were necessary to meet the targeted performance, yielding an average flux of 24.0 LMH. The feed pressure was 11.3 bar, and due to head loss, the final concentrate pressure decreased to 6.53 bar. The black dots in the figure indicate that there are more membrane modules in actuality, which are omitted from the figure for brevity.

The permeate flow rate was set to 64 m^3^/d to match the concentration ratio of 12.5 observed in the FO process with the DSSD. Under these conditions, the specific energy consumption was calculated to be 0.58 kWh/m^3^, which is approximately 4.23 times higher than that of the FO process (0.137 kWh/m^3^).

These results indicate that the FO process employing DSSD can achieve a comparable level of concentration performance to the RO process while requiring approximately 76.4% less energy. Although the FO process involves additional complexity related to the regeneration or recovery of the draw solution, it offers significantly improved energy efficiency over conventional RO from a concentration perspective.

The predicted energy consumption of 0.58 kWh/m^3^ for the RO process was consistent with previous reports using the same or similar membrane modules (e.g., BW30HR-440i) under brackish water conditions, as reported by Abkar et al. (2024) [28].

## 4. Discussion

In this study, a DSSD was proposed and validated through experiments and simulations to overcome the inherent limitations of the conventional FO process. These limitations include the reduction in the osmotic driving force caused by DS dilution and FS concentration. The DSSD achieved a concentration factor of 12.5, which was more than 2.5 times higher than that of the conventional serial configuration under identical membrane area and operating conditions. Furthermore, the specific energy consumption was as low as 0.137 kWh/m^3^, demonstrating superior energy efficiency compared to conventional membrane separation processes.

Additionally, a simulation of the RO process under the same feed conditions showed an energy consumption of 0.58 kWh/m^3^, which was approximately 76.4% higher than that of the FO process. This highlights the superior energy efficiency of the FO process, particularly in concentration-focused applications.

In particular, FO processes can operate under low-pressure conditions and are less susceptible to membrane fouling. However, one of the main challenges that remain is the regeneration or post-treatment of the DS. Therefore, future studies should address the treatment strategies and economic feasibility of DS treatment. For example, if seawater is used as the DS, the diluted DS can be treated using a subsequent RO process, enabling low-energy seawater desalination. Nevertheless, this approach requires the FO facility to be located near coastal areas, which may pose geographical constraints for widespread implementation.

To achieve sustainable desalination, it is important to establish target values for specific energy consumption that reflect both technical feasibility and long-term environmental sustainability. Conventional seawater reverse osmosis processes typically consume between 3 and 4 kWh/m^3^, with advanced configurations incorporating energy recovery devices that reduce this value to approximately 2.9 kWh/m^3^ [29]. In contrast, solar-powered FO systems, particularly when integrated with hybrid technologies such as FO–MD (membrane distillation) or FO–MED (multi-effect distillation), have demonstrated significantly lower specific energy consumption values, ranging from 1.1 to 3.57 kWh/m^3^ depending on the configuration and draw solute regeneration methods applied [30]. However, certain approaches, such as thermal regeneration using solar ponds, still require a much higher energy input (e.g., up to 107 kWh/m^3^), underscoring the importance of careful selection of draw solutes and optimization of thermal energy storage systems [30]. Similarly, multistage solar–thermal desalination processes benefit from latent heat recovery and achieve enhanced thermal efficiency but require optimization of capital cost and heat recovery strategies to ensure scalability [31]. Based on current evidence, an attainable specific energy consumption target of below 2 kWh/m^3^ appears to be a realistic benchmark for small-to medium-scale sustainable desalination systems that leverage solar integration and hybrid processing [30,31].

In summary, this study demonstrated the effectiveness of the DSSD as a novel design strategy for simultaneously enhancing the concentration performance and energy efficiency of the FO process. The results also highlight the potential applicability of this high-efficiency dewatering and resource recovery approach in a wide range of industrial sectors.

## Data Availability

The data presented in this study are available upon request from the corresponding author.

## Abbreviations

The following abbreviations are used in this manuscript:

FO	Forward osmosis
FS	Feed solution
DS	Draw solution
DSSD	Draw solution split distribution
RO	Reverse osmosis
ICP	Internal concentration polarization
ECP	External concentration polarization
NRMSE	Normalized root mean square error
HP	High-pressure pump
LP	Low-pressure pump
BP	Booster pump
ERD	Energy recovery device
MD	Membrane distillation
MED	Multi-effect distillation
A	Water permeability
B	Slat permeability
Δ*π*_ef_	Effective osmotic pressure difference
Δ*P*	Transmembrane pressure
*S*	Structural parameter
*Sh*	Sherwood number
*Re*	Reynolds number
*Sc*	Schmidt number
*d_H_*	Hydraulic diameter
*Q_c_*	Flow rate of concentrated feed solution
*C_c_*	Concentration of concentrated feed solution
*P_c_*	Pressure of concentrated feed solution
*Q_d_*	Flow rate of draw solution
*C_d_*	Concentration of draw solution
*P_d_*	Pressure of draw solution
*Q_dd_*	Flow rate of diluted draw solution
*C_dd_*	Concentration of diluted draw solution
*P_dd_*	Pressure of diluted draw solution
